# Characterizing Awareness of Pre-Exposure Prophylaxis for HIV Prevention in Manila and Cebu, Philippines: Web-Based Survey of Filipino Cisgender Men Who Have Sex With Men

**DOI:** 10.2196/24126

**Published:** 2022-01-07

**Authors:** Arjee Restar, Anthony Surace, Alexander Adia, William Goedel, Adedotun Ogunbajo, Harry Jin, Alberto Edeza, Laufred Hernandez, Susan Cu-Uvin, Don Operario

**Affiliations:** 1 Department of Behavioral and Social Sciences Brown University School of Public Health Providence, RI United States; 2 Brown University Global Health Initiative The Philippine Health Initiative for Research, Service, and Training Providence, RI United States; 3 amFAR, The Foundation of AIDS Research Washington, DC United States; 4 Department of Epidemiology Bloomberg School of Public Health Johns Hopkins University Baltimore, MD United States; 5 Department of Epidemiology School of Public Health University of Washington Seattle, WA United States; 6 Department of Epidemiology Brown University School of Public Health Providence, RI United States; 7 Department of Behavioral Sciences University of Philippines in Manila Manila Philippines; 8 Providence-Boston Center for AIDS Research Providence, RI United States; 9 Department of Medicine Miriam Hospital Providence, RI United States

**Keywords:** HIV prevention, PrEP, men who have sex with men, the Philippines

## Abstract

**Background:**

The Philippines is experiencing an HIV crisis and is considering implementing pre-exposure prophylaxis (PrEP) as a national public health strategy for HIV prevention for cisgender men who have sex with men (cis-MSM). However, critical information on the awareness of PrEP among cis-MSM is needed to roll out this public health initiative.

**Objective:**

This study aims to assess PrEP awareness and related correlates (ie, sociodemographic variables, social factors, and health care access and use) among Filipino cis-MSM.

**Methods:**

We conducted a web-based survey with Filipino cis-MSM (n=179) residing in the cities of Manila and Cebu, Philippines. Multivariable analysis procedures were performed to examine the factors associated with PrEP awareness.

**Results:**

Our sample demonstrated high awareness (134/179, 74.9%) and interest (159/179, 88.8%) in taking PrEP. The adjusted model showed that greater odds of PrEP awareness were associated with having a college education or higher versus a high school education or lower (adjusted odds ratio [aOR] 7.30, 95% CI 1.01-52.47), earning between PHP 10,000 (US $198.6) and PHP 20,000 (US $397.2) versus <PHP 10,000 (US $198.60; aOR 9.32, 95% CI 1.41-6.22), having had a prior HIV test (aOR 6.06, 95% CI 1.20-13.55), having high HIV knowledge (aOR 3.50, 95% CI 1.11-10.98), and having friends who discussed PrEP (aOR 11.17, 95% CI 2.73-14.5).

**Conclusions:**

Our findings demonstrate that Filipino cis-MSM are aware of and interested in taking PrEP, but there is currently an unmet need for such biomedical HIV prevention technologies among this population. Incorporating PrEP education into routine HIV screening and leveraging cis-MSM social networks may be useful in optimizing potential PrEP implementation in the Philippines.

## Introduction

### Background

Globally, cisgender men (ie, individuals who identify their gender as men and are assigned the male sex at birth) who have sex with men (cis-MSM) remain highly vulnerable to HIV infection [1,2]. A meta-analysis found that the HIV prevalence among cis-MSM in the South and Southeast Asia region was 15% [2]. The Philippines, situated in Southeast Asia, is undergoing a major public health crisis as a result of the rapid increase in new HIV cases documented in the past decade. The number of people diagnosed with HIV in the Philippines has increased more than 4-fold, from 16,000 cases in 2010 to 68,000 in 2017 [3] and is concentrated among young, cis-MSM [4]. Of the 6011 new cases of HIV in 2014, 85% were attributed to male-to-male sexual contact with a median age at diagnosis of 28 years [4,5]. The major drivers of HIV infection among Filipino cis-MSM include receptive anal intercourse and sex under the influence of alcohol and other substances [5,6]. Use of HIV prevention services among this population remains low; in a survey of 531,000 cis-MSM in the Philippines, only half reported using condoms during their last sexual encounter [3], and HIV testing rates were lower among cis-MSM compared with those in China and Vietnam [7].

Pre-exposure prophylaxis (PrEP) is an evidence-based, effective biomedical HIV prevention strategy that significantly reduces HIV transmission risk [8]. Currently, PrEP is available as a daily oral pill (eg, Truvada, a combination of emtricitabine and tenofovir), which can reduce the risk of HIV infection by more than 90% when taken as prescribed [9,10]. To date, there is a single ongoing PrEP demonstration project among cis-MSM in the Philippines [11], and the Philippines Department of Health is considering approval of PrEP for public use. Examining levels of PrEP awareness and interest among cis-MSM in the Philippines will inform future rollout and scaling of PrEP programs, especially for vulnerable groups such as cis-MSM.

Awareness of and interest in taking PrEP are key determinants of successful initiation into the PrEP continuum of care [12,13]. PrEP awareness has been researched among American cis-MSM [14,15], but analogous research in other parts of the world is scant. One of the few published reviews examining awareness and willingness to use PrEP among cis-MSM in low- and middle-income countries found low awareness (16.9%-44.3%) but high willingness to use PrEP for HIV prevention (53.3%-74.8%) [16]. Furthermore, a literature review of HIV research in the Philippines showed that there are only a few empirical studies on HIV prevention among cis-MSM, and none of these studies have explored PrEP [17]. Given the ongoing considerations to support implementation of PrEP in national guidelines in the Philippines, it is vital to assess the current level of PrEP awareness and interest among Filipino cis-MSM. Such data are critical for informing future PrEP implementation programs in the Philippines.

### Objective

To date, no known quantitative study has explored awareness of PrEP among Filipino cis-MSM. There is a need for such research given the high vulnerability of Filipino cis-MSM to HIV infection and the heavy burden of HIV infection among this group. To this end, we carried out a web-based survey assessment to examine the relationship between PrEP awareness among Filipino cis-MSM and sociodemographic variables, social factors (eg, marginalization, cohesion, and participation), indicators of health care experiences and access to HIV services, and PrEP indicators.

## Methods

### Participants and Procedures

The data used in this study were obtained from Project #ParaSaAtin, a web-based study that surveyed key populations (eg, cis-MSM) impacted by the HIV epidemic in the Philippines. Informed by epidemiological data, we focused on Manila or National Capital Region (NCR) and Cebu. Specifically, as identified in the Philippines HIV national surveillance report, these are the top geographical areas where HIV infection is concentrated in the Philippines [4]. The purpose of the survey was to examine the structural, social, and behavioral factors that impact the use of HIV/AIDS prevention services, such as PrEP, condoms, and HIV testing. This analysis focused specifically on PrEP awareness and willingness to take PrEP as outcomes of interest.

Between June 2018 and May 2019, we recruited participants by posting our study survey link on flyers and advertisements through web-based social media sites (eg, Facebook groups and Twitter) of community-based organizations that provided services to communities impacted by the HIV/AIDS epidemic in the Philippines. Participants who clicked on the survey completed a web-based written consent and screening process. Eligibility criteria for participants in this study included self-reporting that they (1) were ≥18 years old, (2) identified as cis-MSM, (3) had condomless anal sex within the past year, (4) currently lived in Manila, NCR or Cebu, (5) demonstrated English comprehension (via a brief cognitive screening form), and (6) provided a web-based written consent to participate in the study. The brief cognitive screening form tested participants’ capacity to understand English by asking participants a series of true or false questions based on the consent form. English is common in the Philippines (as it is one of the national languages) [18] but given that the survey was written in English, we wanted to ensure that the enrolled participants demonstrated sufficient language comprehension. To further ensure survey comprehension, we used the Flesch-Kincaid Reading Level Test [19] to design the survey such that it could be read by someone at the sixth grade level. The average range of time for participants to complete the survey was between 20 and 25 minutes. All participants received a PHP 300 (approximately US $5.85) electronic prepaid mobile load card for their time and completion of the survey.

We also used best practices for conducting web-based survey questionnaires. This included having a series of standard web-based survey protective measures, such as (1) *captcha box*, which provides a web-based challenge-response test that eliminates robots from taking the survey [20,21] and (2) unique IP address programming, which prevents the same participants from taking the survey multiple times by only allowing those with a unique IP address to take the survey [21,22]. Study procedures were deemed appropriate by all 3 local HIV/AIDS community-based organizations that were our local partners for this project and collaborated in the development and implementation of this survey. This study received ethical approval from the Brown University Human Research Protection Program Institutional Review Committee in Providence, Rhode Island.

### Measures

We analyzed the correlates of PrEP awareness and interest using a series of questions on sociodemographic factors, social factors (eg, marginalization, cohesion, and participation), and indicators of health care experiences and access to HIV services among the participants.

#### Sociodemographics

We adapted sociodemographic items from the Philippines National Demographic and Health Survey [23]. With the exception of sexual identity, these items included age, current living location, education attainment, past year income, and religious affiliation.

#### Social Marginalization, Cohesion, and Participation Variables

We asked the participants about their social experiences, including marginalization, cohesion (ie, perception of mutual aid, trust, and support) [24], and participation in social events and activities.

For social marginalization, we asked if they had ever been homeless (*yes or no*), were currently unemployed (*yes or no*), and had engaged in sex work within the past 4 months (*yes or no*).

To measure social cohesion, we adapted a 9-item social cohesion scale [24] to specifically refer to cis-MSM communities (Cronbach α=.92). Examples of these items are *You can count on your cis-MSM friends if you need to talk about your problems* and *You can count on your cis-MSM friends to accompany you to the doctor or hospital*. Each item had 5-level Likert response options, from *strongly disagree* (1) to *strongly agree* (5) and was summed (median 22; range 9-45). Similar to previous researchers’ work on this scale [24-26], scores were dichotomized into low versus high levels of social cohesion at the median.

We asked about the engagement of the participants in general community events and activities (ie, general social participation), such as activities related to church, community, or cultural events as well as lesbian, gay, bisexual, and transgender (LGBT)-specific activities (ie, LGBT-specific social participation), such as joining pageants, HIV advocacy groups, and local LGBT organizations. To assess gender- and LGBT-specific social participation, we adapted the social participation scale (Cronbach α=.76 and .75, respectively) [25]. Keeping in-line with previous researchers [25,26], responses were either *yes* or *no* and were summed (median 1, range 0-4 for general social participation and median 3, range 0-5 for LGBT-specific social participation) and then dichotomized into low versus high levels of participation at the median.

#### Health Care and HIV Service Indicators

We assessed participants’ health care experience by asking whether they had current health insurance (*yes or no*) and had ever been discriminated against because of sexual identity (*yes or no*). We used the health care accessibility scale by Haggerty et al [27] to measure our participants’ health care accessibility (Cronbach α=.89). Items included questions about how convenient their provider’s office is with regard to office hours for appointments, duration of next open appointments, waiting time before appointments, traveling to and from the office, and ability to reach the provider when needed. Responses were scored (median 19, range 6-30) and dichotomized into poor or fair versus good or excellent health care accessibility at the median.

We assessed the experiences of access to HIV services, by asking the participants if they had ever had an HIV test. We also asked if they avoided seeking HIV services because of the cost of services, distance of travel to and from the health care facility, sexual identity, and lack of facility-level policy on LGBT antidiscrimination. To assess participant’s HIV knowledge, we used the International AIDS Questionnaire (Cronbach α=.80; median 40, range 23-74), and dichotomized the scores into high versus low HIV knowledge at the median [28].

#### PrEP Indicators

We adapted 3 items on PrEP awareness, interest, and discussion among friends from the study by Restar et al [29]. Specifically, we asked the participants whether they had heard of PrEP (*yes or no*). Regardless of their previous PrEP awareness, all participants then received a brief educational statement about PrEP [30]:

One way to fight HIV is called PrEP, which stands for pre‐exposure prophylaxis. PrEP works by giving HIV‐negative people HIV drugs to keep them from getting HIV. The following questions are about your thoughts and opinions of PrEP.

We then asked if they were interested in taking PrEP (*not interested at all* vs *somewhat or very interested*). We also asked the participants to rate how true the following statement is—*My friends talk about PrEP*—with response options as *very or somewhat true* versus *not true at all*. Participants who were not interested in PrEP were subsequently asked to enter their primary reason for being uninterested in PrEP.

### Analysis

Descriptive analyses were performed to determine proportions and chi-square tests and bivariable and multivariable logistic regression modeling were performed to determine associations among participants. Before running the regression procedures, we performed a sensitivity analysis to determine internal consistency for all of our scale variables (eg, health care accessibility, social cohesion and participation, and HIV knowledge). Multicollinearity tests using the standard procedure for assessing the variance inflation factor were performed to ensure that our independent variables were not collinear. Our results determined that collinearity was not present in our data (all variance inflation factors <4.00) [31]. Logistic regression procedures were performed to determine associations between PrEP awareness and sociodemographics, experiences of social marginalization, social cohesion and participation, health care, access to HIV services, and other PrEP indicators. We then descriptively examined the reasons for not having interest in PrEP. All analyses were performed using Stata standard edition version 15.1 (StataCorp LLC) [32]. Statistical significance was set at 2-sided *P*<.05.

## Results

### Sample Characteristics

A total of 179 cis-MSM completed the survey. Study sample characteristics are presented in (Table 1).

**Table 1:** Study sample characteristics of Filipino cismen who have sex with men (n=179).

Characteristics				Total, n (%)		PrEP^a^-aware (n=134), n (%)		*P* value^b^
**Demographics**								
	**Age**							.08
		18-24	40 (23)		32 (24.2)			
		25-29	59 (33.9)		45 (34.1)			
		30-34	36 (20.7)		31 (23.5)			
		>35	39 (22.4)		24 (18.2)			
	**Current living location**							.52
		Metro Manila or National Capital Region	145 (81)		110 (82.1)			
		Central Visayas	34 (19)		24 (17.9)			
	**Highest educational attainment**							.10
		High school or lower	23 (12.9)		13 (9.7)			
		Some college	34 (19.1)		26 (19.6)			
		College or higher	121 (68)		94 (70.7)			
	**Past year income**							.27
		<PHP 10,000 (US $198.60)	25 (19.6)		21 (15.7)			
		PHP 10,000 (US $198.60)-PHP 20,000 (US $397.20)	38 (21.2)		30 (22.4)			
		PHP 20,000 (US $397.20)-PHP 30,000 (US $ 596.00)	30 (16.8)		23 (17.2)			
		>PHP 30,000 (US $ 596.00)	60 (33.5)		47 (35)			
		No income in the past year	16 (8.9)		13 (9.7)			
	**Religious affiliation**							.14
		Catholic	144 (80.4)		105 (78.4)			
		Non-Catholic (eg, Protestant, Christian)	24 (13.4)		18 (13.4)			
		Nonreligious	11 (6.2)		11 (8.2)			
	**Sexual orientation**							.048^c,d^
		Gay	95 (53)		76 (56.7)			
		Bisexual	71 (39.7)		52 (38.8)			
		Straight	11 (6.2)		5 (3.7)			
		Not listed	2 (1.1)		1 (0.8)			
**Social marginalization, cohesion, and participation**								
	**Ever homeless**							.49
		No	158 (88.3)		117 (87.3)			
		Yes	21 (11.7)		17 (12.7)			
	**Recent (<4 months) sex work engagement**							.79
		No	153 (85.5)		114 (85.1)			
		Yes	26 (14.5)		20 (14.9)			
	**Currently unemployed**							.18
		No	46 (25.7)		31 (23.2)			
		Yes	133 (74.3)		103 (76.8)			
	**Social cohesion**							.24
		Low	82 (45.8)		58 (43.3)			
		High	97 (54.2)		76 (56.7)			
	**General social participation**							.09
		Low	92 (51.4)		64 (47.8)			
		High	87 (48.6)		70 (52.2)			
	**LGBT^e^-specific social participation**							.002
		Low	109 (60.9)		73 (54.5)			
		High	70 (39.1)		61 (45.5)			
**HIV and other health care indicators**								
	**Current health insurance**							.32
		No	88 (49.2)		63 (47)			
		Yes	91 (50.8)		71 (53)			
	**Health care discrimination because of sexual identity**							.03^c,d^
		No	153 (85.5)		110 (82.1)			
		Yes	26 (14.5)		24 (17.9)			
	**Health care accessibility**							.57
		Poor or fair	93 (52)		68 (50.8)			
		Good or excellent	86 (48)		66 (49.2)			
	**Ever had an HIV test**							<.001
		No	37 (20.7)		17 (12.7)			
		Yes	142 (79.3)		117 (87.3)			
	**Avoided HIV services because of the cost of services**							.006
		No	107 (59.8)		88 (65.7)			
		Yes	72 (40.2)		46 (34.3)			
	**Avoided HIV services because of distance of travel to and from the health care facility**							.70
		No	107 (59.8)		79 (59)			
		Yes	72 (40.2)		55 (41)			
	**Avoided HIV services because of sexual identity**							.04
		No	129 (72.1)		102 (76.1)			
		Yes	50 (27.9)		32 (23.9)			
	**Avoided HIV services because of lack of LGBT antidiscrimination policy**							.03
		No	143 (79.9)		112 (83.6)			
		Yes	36 (20.1)		22 (16.4)			
	**HIV knowledge**							.004
		Low	78 (43.6)		50 (37.3)			
		High	101 (56.4)		84 (62.7)			
**PrEP-related indicators**								
	**PrEP discussion among friends**							<.001
		No	103 (57.5)		63 (47)			
		Yes	76 (42.5)		71 (53)			
	**PrEP interest**							.57
		Not at all	20 (11.2)		16 (11.9)			
		Very or somewhat	159 (88.8)		118 (88.1)			

^a^PrEP: pre-exposure prophylaxis.

^b^Chi-square test.

^c^Fisher exact test. Comparison groups for both tests were pre-exposure prophylaxis–aware versus pre-exposure prophylaxis–unaware.

^d^*P*<.05.

^e^LGBT: lesbian, gay, bisexual, and transgender.

Overall, most of the participants currently lived in Manila or NCR (145/179, 81%), had at least college education (121/179, 67.6%), self-identified as Catholic (144/179, 80.4%), and self-identified as gay (95/179, 53%). Approximately one-third of the participants earned PHP 30,000 or more a year (60/179, 33.5%) and were between ages 25 and 29 years (59/179, 33.9%). Most had never experienced homelessness (158/179, 88.3%) and had not recently been involved in sex work (153/179, 85.5%), but most were currently unemployed (133/179, 74.3%). In addition, approximately half of the participants displayed high social cohesion (97/179, 54.2%), showed high general social participation (87/179, 48.6%), exhibited low LGBT-specific social participation (109/179, 60.9%), and reported poor or fair health care accessibility (93/179, 52%).

Half of the participants reported having no current health insurance (88/179, 49.2%), and 14.5% (26/179) reported having experienced health care discrimination because of their sexual identity. For HIV indicators, most of the participants had taken an HIV test (142/179, 79.3%), and less than half had avoided HIV services because of the cost of services (72/179, 40.2%), distance of travel to and from the health care facility (72/179, 40.2%), sexual identity (50/179, 27.9%), and lack of LGBT antidiscrimination policy (36/179, 20.1%). Over half of the participants demonstrated having high HIV knowledge (101/179, 56.4%).

Most of the participants were aware of PrEP (134/179, 74.9%) and were interested in taking PrEP (159/179, 88.8%), and approximately half discussed PrEP among friends (76/179, 42.5%). In total, 11.7% (20/179) of Filipino cis-MSM indicated that they were not interested in taking PrEP and provided their primary reasons for not being interested. Of this subsample, the most common reasons for not being interested in PrEP were the *need to know more information before taking PrEP* (5/20, 25%) and *don’t like taking pills* (5/20, 25%).

### Bivariable Logistic Regression Findings

Findings from bivariable logistic regression on the correlates of PrEP awareness are presented in Multimedia Appendix 1.

Factors that were associated with greater odds of PrEP awareness included (*P*<.05 in all instances) the following: having attained college education or higher versus high school education or lower (odds ratio [OR] 2.67, 95% CI 1.05-6.77), having high LGBT-specific social participation (OR 3.34, 95% CI 1.49-7.48), having had an HIV test (OR 5.50, 95% CI 2.53-11.98), having high HIV knowledge (OR 2.76, 95% CI 1.37-5.55), and having discussed PrEP among friends (OR 9.01, 95% CI 3.35-24.25).

Factors that were associated with lower odds of PrEP awareness included (*P*<.05 in all instances) the following: being straight-identified (OR 0.20, 95% CI 0.05-0.75) and having experienced health care discrimination because of sexual identity (OR 0.21, 95% CI 0.04-0.94). In addition, avoiding HIV services because of cost (OR 0.38, 95% CI 0.19-0.76), sexual identity (OR 0.47, 95% CI 0.22-0.96), and lack of LGBT antidiscrimination policy (OR 0.43, 95% CI 0.19-0.94) were all associated with lower odds of PrEP awareness.

### Multivariable Logistic Regression Findings

The findings from the multivariable logistic regression model are presented in Multimedia Appendix 1. Significantly greater odds of PrEP awareness were found among Filipino cis-MSM who had attained college education or higher versus high school education or lower (adjusted OR [aOR] 7.30, 95% CI 1.01-52.47) and earned between PHP 10,000 and PHP 20,000 versus <PHP 10,000 (aOR 9.32, 95% CI 1.41-6.22). Having ever had an HIV test was associated with approximately 6 times the odds of PrEP awareness (aOR 6.06, 95% CI 1.20-13.55) compared with not having had an HIV test. Those who displayed high HIV knowledge had more than 3 times the odds of being aware of PrEP compared with those with low HIV knowledge (aOR 3.50, 95% CI 1.11-10.98). Filipino cis-MSM who discussed PrEP among friends had greater odds of PrEP awareness (aOR 11.17, 95% CI 2.73-14.50) compared with those who did not.

Significantly lower odds of PrEP awareness were associated with age and location. Specifically, participants aged 25-29 years (aOR 0.15, 95% CI 0.02-0.90) and ≥35 years (aOR 0.11, 95% CI 0.01-0.84) had lower PrEP awareness compared with 18- to 24-year-old participants. Participants living in Cebu had lower odds of PrEP awareness (aOR 0.83, 95% CI 10.70-0.98) compared with those living in Metro Manila or NCR region.

## Discussion

### Overview

This paper offers insights on the potential of PrEP as an HIV prevention strategy among cis-MSM in the Philippines, a country with one of the fastest growing HIV crises in the world. Currently, the Philippines does not have an established PrEP program and is still in the nascent stages of rollout with only 1 current PrEP demonstration project. This follows broader trends in Asia, where few countries have implemented PrEP programs [33]. Insights in this paper point to a growing need and demand for PrEP in the Philippines for populations highly impacted by HIV, such as cis-MSM populations. The potential for the rollout of PrEP-based interventions and programming is contingent on public health efforts to increase levels of PrEP awareness of and interest in taking PrEP among cis-MSM in the Philippines.

Overall, PrEP awareness and interest in taking PrEP were high in our sample, as most participants indicated that they were both aware of and interested in PrEP. There have been few efforts to document PrEP awareness and interest across the Asia-Pacific region; what little research exists suggests that knowledge about PrEP is low in the region [33]. Our findings, however, contradict such research, as we found that most participants were aware of PrEP and had a high degree of PrEP knowledge. These results may have been because of our use of community-based organizations, which had HIV-specific programming and content, for recruitment. Moreover, previous research has found associations between high HIV knowledge and having access to and better engagement with HIV services [34,35]. In our sample, HIV knowledge was associated with greater odds of PrEP awareness. As such, it is plausible that Filipino cis-MSM may gain HIV knowledge and awareness of PrEP through their connections to HIV prevention services. However, further research is needed to elucidate this connection. Ultimately, this points to a potential role for community-based organizations in generating demand for and education on PrEP, a role that organizations in other settings have been willing and able to take.[36]. Indeed, Republica Act 11166, the new HIV policy recently passed in the Philippines, explicitly calls out the need for policy makers, practitioners, and scientists to work alongside such organizations in building a stronger HIV response [37]. Given these findings, a key policy step in building PrEP rollout in the Philippines could be the integration of community-based organizations as partners in the strategic planning and implementation processes from the very start of the national PrEP program.

The results from our multivariable model also imply the means by which PrEP implementation may be optimized. First, the association between having ever had an HIV test and awareness of PrEP is positive, as it demonstrates the potential linkage of PrEP education with routine health care, such as HIV tests. Prior research has argued that this routinization of PrEP discussions in the provision of other health services both destigmatizes PrEP and facilitates broader PrEP awareness [38]. As the Philippines continues to design the implementation of its own PrEP program, the integration of the burgeoning PrEP program alongside the continued expansion of HIV testing and service provision could yield the strongest uptake.

Second, the finding that discussion of PrEP with friends was linked to greater awareness of PrEP points to how social networks may be leveraged to improve PrEP uptake. Prior research has noted that social networks may play a role in promoting PrEP awareness [39]. However, research has also shown that MSM social networks may be a source of PrEP stigma—namely that PrEP is associated with sexual promiscuity and sexually transmitted infections [40]. Such stigma may serve as a barrier to PrEP education and use [41]. Although we did not specifically assess the nature of the discussions about PrEP in this study, future research should aim to do so to gauge the feasibility of using extended networks of cis-MSM to spread PrEP awareness and increase demand and use. Questions about the directionality of how knowledge is passed on (eg, whether having awareness of PrEP may lead to more discussion among friends about PrEP or whether having friends who discuss PrEP may lead to increased awareness of PrEP) and how individuals seek information about PrEP among friends who are also informed about PrEP within their social circles remain unanswered. Qualitative research may be especially useful in exploring this, as it may allow for a deeper understanding of the complex dynamics of social interaction around PrEP. Such research may shed light on how discussing PrEP with friends impacts awareness and desire to use PrEP.

Finally, it is worth noting the other correlates of PrEP awareness levels that we found. PrEP awareness in this sample was significantly lower among those over the age of 25 years (compared with those who were younger), who had very low income and education levels (compared with those with moderate to high income or education), and who resided in Cebu (compared with Manila). These results suggest that there are distinct health disparities across regions and socioeconomic strata within the population of cis-MSM in the Philippines. In addition to sociodemographics, it is important for future research in this area to better understand the sexual behaviors and relationship contexts (eg, casual vs romantic, long-term vs short-term, and monogamous vs having more than one partner) of cis-MSM who are unaware of PrEP, as PrEP may likely be beneficial to them and their partners. Such research can better inform PrEP campaigns aimed at promoting equity across cis-MSM populations. As such, there is a need for continually monitoring the reach of PrEP education and communication campaigns to mitigate such disparities within the country. Informational campaigns must particularly acknowledge differences in language or dialect, region, and literacy levels as potential barriers to PrEP awareness.

### Principal Findings

Filipino cis-MSM are aware of and interested in taking PrEP. Those who were less aware of PrEP tended to come from poorer backgrounds and have less education. In addition, those who had encountered barriers to health care (ie, costs of medical visits or discrimination from health care staff) were less likely to be aware of PrEP. Incorporating PrEP education into routine HIV screening and leveraging cis-MSM social networks may be useful in increasing PrEP awareness and uptake.

### Limitations

This study has several limitations worth noting. First, as we relied on sampling through community-based organizations, our sample is unlikely to be representative of the cis-MSM populations and therefore, our findings cannot be generalized to all cis-MSM in the Philippines, particularly those who may be at higher risk for HIV. That is, although all enrolled participants reported condomless anal sex in the past year, our participants come from 2 urban areas and most of them were educated (ie, approximately 121/179, 67.6% had completed college education or above and 34/179, 19% had some college education) and therefore, may be more aware of HIV than those who do not have access to community-based organizations that offer HIV-related programs. As such, our findings may not accurately reflect the population of high-risk cis-MSM. This is underscored by the high level of HIV knowledge in our sample. Second, we recruited participants from major metropolitan areas in the Philippines, so our findings likely do not capture awareness and education in other regions (eg, rural areas). Finally, our survey was only offered in English; although English is a national language of the Philippines, those only comfortable communicating in Tagalog (or other dialects in the Philippines) were screened out. These individuals may differ significantly from our study sample (eg, comfort communicating only in Tagalog may reflect lower access to education) in ways that may have impacted our results.

### Conclusions

Our research represents the first effort to characterize PrEP awareness and willingness to take PrEP among a sample of Filipino cis-MSM. Our findings demonstrate that Filipino cis-MSM are aware of and interested in taking PrEP, but there is currently an unmet need for such biomedical HIV prevention technologies among this population. However, it must also be acknowledged that participants in our sample were likely to be engaged with HIV prevention services. More work is needed to identify the needs of less visible Filipino cis-MSM (ie, those disconnected from HIV prevention services who may be underserved by the national health care system of the Philippines). This study contributes to the scant research on this population and offers insights on the potential of PrEP as a means to reduce HIV among this population.

## Appendix

Multimedia Appendix 1 Bivariable and multivariable logistic regression models.

## Abbreviations

aOR: adjusted odds ratio
cis-MSM: cisgender men who have sex with men
LGBT: lesbian, gay, bisexual, and transgender
NCR: National Capital Region
NIH: National Institutes of Health
OR: odds ratio
PrEP: pre-exposure prophylaxis
