# Supplementary material for: Characterizing Awareness of Pre-Exposure Prophylaxis for HIV Prevention in Manila and Cebu, Philippines: Web-Based Survey of Filipino Cisgender Men Who Have Sex With Men
Source: J Med Internet Res. 2022 Jan 7;24(1):e24126. doi: 10.2196/24126 (PMC8783281; doi:10.2196/24126)
Supplement: Multimedia Appendix 1 [file jmir_v24i1e24126_app1.docx]

**Appendix 1.** Bivariable and multivariable logistic regression models.

| Characteristics | | | PrEP^a^ awareness | | | |
| --- | --- | --- | --- | --- | --- | --- |
|  | | | Unadjusted model | | Adjusted model^b^ | |
|  | | | OR^c^ (95% CI) | *P* value | aOR^d^ (95% CI) | *P* value |
|  | | | | | | |
| **Sociodemographic** | | | | | | |
|  | **Age (years)** | | | | | |
|  |  | 18-24 | Reference | N/A^e^ | Reference | N/A |
|  |  | 25-29 | 0.80 (0.30-2.14) | .66 | 0.15 (0.02-0.90) | .04^f^ |
|  |  | 30-34 | 1.55 (0.45-5.25) | .48 | 0.25 (0.03-1.95) | .19 |
|  |  | >35 | 0.40 (0.14-1.09) | .08 | 0.11 (0.01-0.84) | .03^f^ |
|  | **Current living location** | | | | | |
|  |  | Metro Manila or National Capital Region | Reference | N/A | Reference | N/A |
|  |  | Cebu | 0.97 (0.88-1.06) | .52 | 0.83 (0.70-0.98) | .03^f^ |
|  | **Highest educational attainment** | | | | | |
|  |  | High school or lower | Reference | N/A | Reference | N/A |
|  |  | Some college | 2.50 (0.79-7.84) | .12 | 2.43 (0.33-17.7) | .38 |
|  |  | College or higher | 2.67 (1.05-6.77) | .04^f^ | 7.30 (1.01-52.47) | .048^f^ |
|  | **Past year income** | | | | | |
|  |  | <PHP 10,000 (US $198.60) | Reference | N/A | Reference | N/A |
|  |  | PHP 10,000 (US $198.60)-PHP 20,000 (US $397.20) | 2.50 (0.89-7.01) | .08 | 9.32 (1.41-61.22) | .02^f^ |
|  |  | PHP 20,000 (US $397.20)-PHP 30,000 (US $ 596.00) | 2.19 (0.74-6.47) | .16 | 2.49 (0.40-15.48) | .33 |
|  |  | >PHP 30,000 (US $ 596.00) | 2.41 (0.96-6.00) | .06 | 3.95 (0.67-23.28) | .13 |
|  |  | <PHP 10,000 (US $198.60) | 2.88 (0.76-12.02) | .15 | 9.94 (0.73-13.46) | .08 |
|  | **Religious affiliation** | | | | | |
|  |  | Catholic | Reference | N/A | Reference | N/A |
|  |  | Non-Catholic (eg, Protestant, Christian) | 1.11 (0.41-3.01) | .83 | 0.40 (0.07-2.13) | .28 |
|  |  | Nonreligious | 1.00 (0.99-1.01) | .81 | 1.34 (0.43-4.19) | .61 |
|  | **Sexual orientation** | | | | | |
|  |  | Gay | Reference | N/A | Reference | N/A |
|  |  | Bisexual | 0.68 (0.33-1.41) | .31 | 0.59 (0.16-2.18) | .44 |
|  |  | Straight | 0.20 (0.05-0.75) | .02^f^ | 0.09 (0.00-1.40) | .09 |
|  |  | Not listed | 0.25 (0.01-4.18) | .34 | 0.09 (0.00-4.99) | .59 |
| **Social marginalization, cohesion, and participation** | | | | | | |
|  | **Ever homeless** | | | | | |
|  |  | No | Reference | N/A | Reference | N/A |
|  |  | Yes | 1.48 (0.47-4.68) | .50 | 6.60 (0.76-56.77) | .09 |
|  | **Currently unemployed** | | | | | |
|  |  | No | Reference | N/A | Reference | N/A |
|  |  | Yes | 0.60 (0.28-1.25) | .18 | 0.34 (0.07-1.65) | .19 |
|  | **Recent (<4 months) sex work engagement** | | | | | |
|  |  | No | Reference | N/A | Reference | N/A |
|  |  | Yes | 1.14 (0.42-3.04) | .79 | 0.20 (0.03-1.30) | .09 |
|  | **Social cohesion** | | | | | |
|  |  | Low | Reference | N/A | Reference | N/A |
|  |  | High | 1.49 (0.76-2.95) | .24 | 0.53 (0.14-1.94) | .34 |
|  | **General social participation** | | | | | |
|  |  | Low | Reference | N/A | Reference | N/A |
|  |  | High | 1.80 (0.90-3.59) | .10 | 0.64 (0.15-2.77) | .56 |
|  | **LGBT**^g^**-specific social participation** | | | | | |
|  |  | Low | Reference | N/A | Reference | N/A |
|  |  | High | 3.34 (1.49-7.48) | .003^h^ | 3.20 (0.57-17.87) | .18 |
| **HIV and other health care indicators** | | | | | | |
|  | **Current health insurance** | | | | | |
|  |  | No | Reference | N/A | Reference | N/A |
|  |  | Yes | 1.40 (0.71-2.77) | .32 | 0.47 (0.11-1.97) | .31 |
|  | **Health care discrimination because of sexual identity** | | | | | |
|  |  | No | Reference | N/A | Reference | N/A |
|  |  | Yes | 0.21 (0.04-0.94) | .04^f^ | 5.88 (0.81-42.42) | .08 |
|  | **Health care accessibility** | | | | | |
|  |  | Poor or fair | Reference | N/A | Reference | N/A |
|  |  | Good or excellent | 1.21 (0.61-2.39) | .58 | 0.94 (0.24-3.72) | .94 |
|  | **Ever had an HIV test** | | | | | |
|  |  | No | Reference | N/A | Reference | N/A |
|  |  | Yes | 5.50 (2.53-11.98) | <.001^i^ | 6.06 (1.20-13.55) | .03^f^ |
|  | **Avoided HIV services because of the cost of services** | | | | | |
|  |  | No | Reference | N/A | Reference | N/A |
|  |  | Yes | 0.38 (0.19-0.76) | .006^h^ | 1.65 (0.42-6.41) | .46 |
|  | **Avoided HIV services because of distance of travel to and from the health care facility** | | | | | |
|  |  | No | Reference | N/A | Reference | N/A |
|  |  | Yes | 1.14 (0.57-2.29) | .70 | 1.00 (0.25-3.96) | .99 |
|  | **Avoided HIV services because of sexual identity** | | | | | |
|  |  | No | Reference | N/A | Reference | N/A |
|  |  | Yes | 0.47 (0.22-0.96) | .04^f^ | 0.89 (0.18-4.38) | .89 |
|  | **Avoided HIV services because of lack of LGBT antidiscrimination policy** | | | | | |
|  |  | No | Reference | N/A | Reference | N/A |
|  |  | Yes | 0.43 (0.19-0.94) | .04^f^ | 1.46 (0.27-7.83) | .66 |
|  | **HIV knowledge** | | | | | |
|  |  | Low | Reference | N/A | Reference | N/A |
|  |  | High | 2.76 (1.37-5.55) | .004^h^ | 3.50 (1.11-10.98) | .03^f^ |
| **PrEP-related indicators** | | | | | | |
|  | **PrEP discussion among friends** | | | | | |
|  |  | No | Reference | N/A | Reference | N/A |
|  |  | Yes | 9.01 (3.35-24.25) | <.001^i^ | 11.17 (2.73-14.50) | .001^i^ |
|  | **PrEP interest** | | | | | |
|  |  | Not at all | Reference | N/A | Reference | N/A |
|  |  | Very or somewhat | 1.38 (0.43-4.39) | .58 | 0.65 (0.07-6.13) | .71 |

^a^PrEP: pre-exposure prophylaxis.

^b^Model is adjusted for all variables.

^c^OR: odds ratio.

^d^aOR: adjusted odds ratio.

^e^N/A: not applicable.

^f^*P*<.05.

^g^LGBT: lesbian, gay, bisexual, transgender, queer.

^h^*P*<.01.

^i^*P*<.001.
